# Clinical Remarks on Surgical Cases in the Buffalo Hospital of the Sisters of Charity

**Published:** 1872-11

**Authors:** J. F. Miner


ART. IV.—*Clinical Remarks on Surgical Cases in The Buffalo Hospital of the Sisters of Charity.* By. Prof. J. F. MINER, M. D.

(Reported by EDWARD N. BRUSH.)

Case I. *Ligation of the Femoral Artery.* Patrick Leary, aged 36 was admitted to the Hospital Sept. 19, 1872. His history is as follows: While passing between two cars they came together, and a large iron bolt similar to the coupling pin was thrust into the anterior and inner portion of the left thigh passing back of the bone completely through the muscular portion. I saw him a few moments after the accident and on making an examination found the condition of the leg as just stated. The wound was largest on the front of the thigh and I could pass two fingers completely through the opening. The bone was not injured, and there was but little bleeding, the wound being a lacerated one.

He was, by my advise, sent to the Hospital where he fell under the care of Dr. Boardman at that time in charge. When I entered upon my term at the Hospital, Oct. 1st, I found the patient doing well, the wound healing, and with the exception of a slight diarrhœa every thing was progressing favorably. Early on the morning of the 2d I was hastily summoned to see the patient, and found that he had been bleeding profusly from the wound.

With the assistance of Dr. Boardman I ligated the Femoral Artery below the Profunda. All hæmorrhage immediately ceased. The patient on recovering from the Anæsthesia seemed quite cheerful although faint from loss of blood. He was placed in bed and tonics and stimulants administered. Every thing progressed favorably, the parts retained their natural color and heat, the process of repair in the original wound went on, and our patient seemed in a fair way of recovering, when in about forty-eight hours from the first hæmorrhage, he began to bleed again. The nurse having been warned of the possibility of such an occurrence was prepared and soon checked the bleeding. On the morning of the 4th with the assistance of Dr. Boardman I again ligated the Femoral this time above the Profunda as the bleeding was now clearly from that vessel.

The patient was again placed in bed, stimulus administered and heat applied to the leg. But Gentlemen, I am sorry to say, every thing we could do for the unfortunate man was of no avail, the leg began to mortify and the patient growing weaker and weaker day after day, at the end of a week died delirious. The question naturally arises, and Gentlemen, it is one which will often arise in your minds; did we do the best thing for our patient, would amputation have afforded him a better chance for his life? You are all of you more or less familiar with the results of amputation of the thigh, you know that it is a serious operation, and that in many instances it is a fatal one.

To have amputated at the first operation would have perhaps been looked upon as unnecessary. To have performed the operation at the time of the second ligation would have probably from the weak and exhausted state of the patient. proved fatal.

In my own mind however the question has arisen as to whether I did right in not amputating in the first instance. We often learn more from our mistakes, than we do from our most brilliant successes, and I leave this case to your consideration, hoping that you may be enabled to profit by it.

Case II. *Necrosis of the Tibia.* Michael Carmody, aged 43 was admitted to the Hospital Oct. 1st 1872, with the following history: On the 28th of July while getting from a train in motion, he received a severe blow upon the tibia producing an abrasion of the skin which resulted in an open ulcer through which at various times spiculæ of bone were discharged.

On being admitted to the hospital an examination revealed necrosed bone, and an operation was determined upon.

Oct. 4th. The patient was put under the influence of chloroform and an incision made down to the bone, which was found necrosed for a space of three inches in length by one in breadth.

By the use of a sharp gouge the dead bone was removed, the parts brought loosly in apposition, and warm water dressing applied. With the exception of a slight hæmorrhage which was easily controlled, every thing progressed favorably and in about five weeks the patient was discharged cured.

CASE III. *Amputation of Thigh.*, Andrew J———, a sailor, aged 21, was admitted to the Marine Ward, in June, with a injury caused by a barrel of flour falling from a height of eight feet upon his knee. He was subsequently transferred to the Surgical ward, then in charge of Dr. Boardman. When he came under my care I found the leg and foot enormously swollen, as you now see. Several openings are seen from which pus is discharged, and on introduction of probe, dead bone is detected near the knee. The nurse says that in moving the leg he has at different times felt a distinct grating sound in the knee joint. I am, however, unable at present to detect any such feeling.

Our patient being placed under the influence of chloroform, I will introduce a silver probe into some of these various sinuses which evidently lead down to the bone. By the aid of the probe I find the femur diseased and roughened as high up as the junction of the lower and middle third. The tibia is also fonnd to be diseased for nearly its whole length.

The question now comes up, what shall we do for our patient. Exsection from the amount of bone implicated is out of the question. Were the bones of the leg simply implicated this procedure might be considered, or was the knee joint alone implicated excision might be substituted in place of amputation.

I have in my possession the shaft of the fibula, which I will show you when I come to talk about exsections which I removed for disease; the patient recovered in a short time, and had good use of his leg. I have also several times removed various portions of the tiabia for disease or injury, and in the majority of cases with a good result. Exsection of any considerable portion of the shaft of the femur, while it is attended with considerable risk, would not, if successful, afford the patient a useful limb. The head and neck of this bone have, however, been removed for disease of the hip joint, and in instances where the patient was not too much exhausted by the disease, with good results. Of excision of the knee joint I cannot speak so favorably. Some of you saw the operation performed in this hospital last winter, for gun-shot injury, with a fatal result.

Prof. Hamilton says that he fully believes that in cases of excision of this joint full fifty per cent. have proved fatal or required subsequent amputation. In cases of excision for gun-shot wounds the mortality is even higher than this.

Amputation, then, is our only alternative, and from the appearance of our patient I have many grave doubts as to its success.

Amputations, gentlemen, as you know, are divided into two classes, primary and secondary; primary being those which are performed immediately after reaction; while a secondary amputation is that which is performed after the part has passed through the various stages of inflammation. It is evident to you all that this is a secondary amputation.

The general mortality in amputations of the thigh in civil practice, ranges from 35 to 50 per cent. The mortality increases in a direct ratio as you approach the trunk.

The causes which induce a fatal result in amputations are shock, hemorrhage, pyæmia, erysipelas, profuse suppuration, gangrene, etc. As to the nature of these causes you will be more fully instructed hereafter.

The patient is now fully under the influence of the chloroform, and I will proceed to amputate the thigh. In order to escape all diseased bone, I will make the section through the middle third, using the flap method. After securing the bleeding vessels, the sharp edges of the bone will be smoothed off by the bone forceps.

The flaps are now carefully sponged off and brought together by silk sutures and supported by adhesive plaster, warm water dressings applied, and the patient returned to his bed. On recovering from the anæsthetic he will be allowed a little brandy and water to stimulate him, and opium to allay pain.

Should the case progress favorably, he will be kept supported, good food given him, and all the proper hygienic measures adopted to prevent, if possible, any of the sequelæ, which often follow such operations.

[Contrary to the general expectation, the patient made a rapid convalescence, and in less than a month was able to be out of bed.]

CASE IV.—*Necrosis of Femur.*—Thomas Ryan, aged 19, comes before you to-day, gentlemen, to have an operation performed for necrosis of the femur.

He has been suffering for upward of two years, but cannot trace the disease to any direct injury. With some of the causes which will induce necrosis or death of bone you are, some of you, familiar; to the others it will be more fully explained hereafter, and need, not on this occasion receive our attention. Suffice it to say that the bone loses its nutrition and dies. After the death of the bone it becomes simply a foreign body, and nature makes an effort to throw it off. Fistulous openings appear over the part affected, through which pus and spiculæ of bone are from time to time discharged.

These openings are plainly to be seen in the case of this young man, and he informs me that small portions of bone have at various times been discharged through some of the many openings which have made their appearance in different portions of the lower third of his thigh.

In the case before you the periosteum has become separated from the bone. New bone has been formed or secreted by the periosteum enclosing the old dead bone, to which the name of a sequestrum is now given.

The discharge produced by the irritaiing action of the old bone, which now acts as a foreign substance, and the pus secreted by the granulations of new bone, find way out through openings in the new bone and periosteum. New bone is not deposited at these points, and they remain open, giving exit to pus and at times to speculæ of bone. These openings in the bone are termed cloacæ. I am able through the cutaneous opening to introduce my probe into one of these cloacæ and immediately come upon dead bone. This is not, however, the only appearance which necrosed bone presents.

The periostium is separated, and death ensuing, the dead portions are immediately thrown off without being inclosed, and if the surface involved is not too great, nature by her efforts may effect a spontaneous cure, otherwise a very slight operation will often be sufficient to relieve the patient.

Syphilis may cause necrosis, and when this is known or suspected to be the fact, the patient should be put upon constitutional treatment before an operation is attempted.

The patient being now fully anæsthetized, I will open down to the bone, following the track of one of these fistulæ. I come immediately upon an opening in the new bone, which being too small to admit of the introduction of my finger or forceps, I will enlarge with the gouge and trephine.

I am now able to introduce my strong forceps into the opening and remove this sequestrum, which lies loose in the cavity. I feel with my fingers and probe various other smaller ones, which are carefully removed until the whole cavity now feels smooth and destitute of all foreign substance. The wound will be brought together and warm water dressings applied.

[This patient progressed favorably for a few days, when symptoms of pyæmia showed themselves, and in about two weeks he died.]

